# Integrating Breathing Techniques Into Psychotherapy to Improve HRV: Which Approach Is Best?

**DOI:** 10.3389/fpsyg.2021.624254

**Published:** 2021-02-15

**Authors:** Patrick R. Steffen, Derek Bartlett, Rachel Marie Channell, Katelyn Jackman, Mikel Cressman, John Bills, Meredith Pescatello

**Affiliations:** Department of Psychology, Brigham Young University, Provo, UT, United States

**Keywords:** HRV, soothing rhythm breathing, 6 breath per minute breathing, psychotherapy, biofeedback

## Abstract

**Introduction:**

Approaches to improve heart rate variability and reduce stress such as breathing retraining are more frequently being integrated into psychotherapy but little research on their effectiveness has been done to date. Specifically, no studies to date have directly compared using a breathing pacer at 6 breaths per minute with compassion focused soothing rhythm breathing.

**Current Study:**

In this randomized controlled experiment, 6 breaths per minute breathing using a pacer was compared with compassion focused soothing rhythm breathing, with a nature video being used as a control group condition.

**Methods:**

Heart rate variability (HRV) measures were assessed via electrocardiogram (ECG) and respiration belt, and an automated blood pressure machine was used to measure systolic diastolic blood pressure, and heart rate (HR). A total of 96 participants were randomized into the three conditions. Following a 5-min baseline, participants engaged in either 6 breath per minute breathing, soothing rhythm breathing, or watched a nature video for 10 min. To induce a stressful state, participants then wrote for 5 min about a time they felt intensely self-critical. Participants then wrote for 5 min about a time they felt self-compassionate, and the experiment ended with a 10-min recovery period.

**Results:**

Conditions did not significantly differ at baseline. Overall, HRV, as measured by standard deviation of NN intervals (SDNN), low frequency HRV (LF HRV), and LF/HF ratio, increased during the intervention period, decreased during self-critical writing, and then returned to baseline levels during the recovery period. High frequency HRV (HF HRV) was not impacted by any of the interventions. The participants in the 6 breath per minute pacer condition were unable to consistently breathe at that rate and averaged about 12 breaths per minute. Time by Condition analyses revealed that both the 6 breaths per minute pacer and soothing breathing rhythm conditions lead to significantly higher SDNN than the nature video condition during breathing practice but there were no significant differences between conditions in response to the self-critical and self-compassionate writing or recovery periods. The 6 breath per minute pacer condition demonstrated a higher LF HRV and LF/HF ratio than the soothing rhythm breathing condition, and both intervention conditions had a higher LF HRV and LF/HF ratio than the nature video.

**Conclusions:**

Although the 6 breath per minute pacer condition participants were not able to breath consistently at the low pace, both the participants attempting to breathe at 6 breaths per minute as well as those in the soothing rhythm breathing condition effectively increased HR variability as measured by SDNN, and attempting to breathe at 6 breaths per minute led to the highest LF HRV and LF/HF ratio. Both breathing approaches impacted HRV more than watching a relaxing nature video and can potentially be used as key adjuncts in psychotherapy to aid in regulating physiological functioning, although it appears that consistent breathing practice would be needed.

## Introduction

Heart rate variability (HRV), an important measure of health and wellbeing, is significantly impacted by mood and mental health and is increasingly being used as a measure of outcome in psychotherapy studies (Steffen et al., 2017; Caldwell and Steffen, 2018; Lehrer, 2018; Fiskum, 2019; Petrocchi and Cheli, 2019). Depression is related to decreased HRV, and psychotherapy potentially can help improve both depression and HRV. Panic disorder patients also show decreased HRV and successful treatment of panic disorder is related to an increase in HRV (Garakani et al., 2009). One approach to directly address HRV is to integrate biofeedback into psychotherapy, which includes breathing exercises that effectively increase HRV (Lehrer and Gevirtz, 2014; Caldwell and Steffen, 2018; Lehrer, 2018). Another approach to psychotherapy, Compassion Focused Therapy, includes soothing rhythm breathing exercises designed to positively impact HRV (Gilbert, 2014). It is not known, however, if these two approaches improve HRV relative to a control condition or if one approach is better than the other. The goal of the current study was to examine these questions directly using a randomized controlled experiment.

### HRV, Health, and Wellbeing

Heart rate variability is the variability in time interval between hearts beats. A common belief is that the amount of time between heart beats is relatively constant. However, in a healthy heart the inter beat interval can be highly variable, and higher variability (higher HRV) is related to better health (Thayer et al., 2012; Shaffer and Venner, 2013; Shaffer et al., 2014). Low HRV, on the other hand, is related to increased morbidity and mortality. In addition to physical health problems impacting heart functioning, HRV is also significantly impacted by mood and mental wellbeing. A large body or research indicates that depression, anxiety, and stress are all related to decreased HRV (Kemp et al., 2010, 2012; Lehrer and Gevirtz, 2014). Depression and anxiety are highly prevalent problems that negatively impact people’s lives across almost all areas of physical and mental health (Bloom et al., 2011; Kessler et al., 2012, 2014). HRV in particular is negatively impacted by depression and anxiety, with parasympathetic functioning impaired.

Because HRV is significantly impacted by mental wellbeing, a number of researchers and psychotherapists are beginning to include HRV both as an outcome measure and a specific target for treatment (Caldwell and Steffen, 2018; Lehrer, 2018; Fiskum, 2019; Petrocchi and Cheli, 2019; Christodoulou et al., 2020). However, even when psychiatric medications effectively improve mental health and reduce symptoms, they do not improve HRV levels (Kemp et al., 2010; Bassett, 2016). One study observed that starting antidepressant medication decreased HRV levels and stopping antidepressant medication usage increased HRV levels (Licht et al., 2010). Psychotherapy has also demonstrated effectiveness in treating depression, but most have not shown improvement in HRV (Hollon et al., 2005; de Matt et al., 2007; Wheeler et al., 2014).

### Breathing, Biofeedback, and HRV

Psychotherapy studies that have shown promise in improving HRV in addition to mental health have included breathing exercises. Chien et al. (2015) compared a traditional cognitive behavior therapy (CBT) approach with CBT combined slow breathing exercises in the treatment of major depression. They found that CBT plus slowing breathing had significant increases in HRV in addition to reducing depressive symptoms, whereas the traditional CBT group did not improve HRV levels. HRV biofeedback, which has been shown to significantly increase HRV levels, also focuses on slow breathing (Lehrer and Gevirtz, 2014). In HRV biofeedback clients learn to breathe at their resonance frequency rate (typically about 6 breaths per minute) using feedback on a computer screen showing their respiration rate and depth, and their heart rate (HR) change in real time. HRV biofeedback improves HRV in general and in clients with depression (Karavidas et al., 2007; Andrasik and Grazzi, 2014; Caldwell and Steffen, 2018).

Karavidas et al. (2007) studied HRV biofeedback as a standalone treatment for major depression and found significant reductions in depression. HRV increased during the treatment but decreased to baseline levels at follow up. Siepmann et al. (2008) also used HRV biofeedback as a treatment for depression and found significant reductions in depression and significant increases in HRV at follow up. Several studies have shown that mindfulness and physical activity, in addition to HRV biofeedback, can increase HRV. For example, Brinkmann et al. (2020) found that HRV biofeedback and mindfulness training both improved overall well-being and HRV. Wyner (2015) also found that adding biofeedback to mindfulness and compassion-based relaxation training leads to better results.

### 6 Breath per Minute Breathing Training and HRV

The theorized mechanisms by which 6 breathe per minute breathing impacts HRV is through stimulating respiratory and autonomic reflexes such as the baroreflexes leading to improved baroreflex function as well as increased gas exchange efficiency (Lehrer, 2018). A number of studies have focused specifically on 6 breath per minute training as a way to improve HRV. Studies by Bernardi et al. (2001), Radaelli et al. (2004), and Chang et al. (2013) found that controlled slow breathing at 6 breaths per minute is associated with increased HRV when compared to natural breathing rates (Russo et al., 2017). Tharion et al. (2012) found that individuals practicing breathing at 6 breaths per minute for 1 month experienced decreased resting breathing rate and increased resting HF HRV. Sürücü et al. (2021) found increased HF and LF HRV and an increased LF/HF ratio in healthy adults that practiced 6 breaths per minute breathing. Van Diest et al. (2014) also determined that breathing at 6 breaths per minute leads to higher HRV than 12 breaths per minute. Lin et al. (2019) studied patients diagnosed with major depressive disorder (MDD) that were either treated with medication for MDD, or with HRV biofeedback group for 60 min a week for 6 weeks in addition to medication. Compared to the medication only patients, the HRV biofeedback plus medication patients had improved depression and anxiety symptom scores as well as improved SDNN, LF HRV, and LF/HF ratio. Interestingly, the HF HRV scores did not significantly change.

### Compassion Focused Therapy and HRV

Paul Gilbert developed Compassion Focused Therapy (CFT) to cultivate compassion for self and others, increase ability to tolerate negative emotions, and live life more fully (Gilbert, 2009, 2014). In creating CFT, Gilbert integrated concepts from a broad range of disciplines including evolutionary psychology, social and developmental psychology, and Buddhist psychology, in addition to traditional CBT approaches. Physiological functioning is also addressed in CFT, and measures such as HRV are recognized as key variables in overall healthy functioning (Kirby et al., 2017).

People who score higher in trait compassion have higher HRV (Svendsen et al., 2016), and those high in self-compassion have higher HRV relative to those low self-compassion in response to the Trier Social Stress Test (Luo et al., 2018). Compassion meditation exercises improve immune responses (Pace et al., 2009). Compassion interventions successfully decrease self-criticism, increase compassion for self and others, as well as increase HRV (Petrocchi et al., 2017; Sommers-Spijkerman et al., 2018; Bello et al., 2020). CFT has been found to reduce the symptoms of depression and anxiety (Judge et al., 2012; Frostadottir and Dorjee, 2019), social anxiety disorder (Gharraee et al., 2018), and improve overall well-being (Bello et al., 2020). Matos et al. (2017) found that compassionate mind training improved positive emotions and compassion for self and others, as well as improving HRV.

### Soothing Rhythm Breathing and Writing Exercises

A key practice in CFT is soothing rhythm breathing. In addition to working on compassion, a key goal of CFT is to also improve HRV and vagal functioning. Soothing rhythm breathing helps accomplish this goal by slowing and deepening an individual’s breathing and encouraging them to focus on slowing the mind the body (Matos et al., 2017). As such, soothing rhythm breathing has been shown to promote calmness by activating the vagal parasympathetic nervous system (Porges, 2007; Matos et al., 2017). Consequently, higher HRV is associated with an increased ability to self-sooth when stressed, which is a desired outcome of CFT (Rockliff et al., 2008).

Matos et al. (2017) found significant improvement in HRV for participants of a non-clinical population in a compassionate mind training intervention that included soothing rhythm breathing. Additionally, Kim et al. (2020) used soothing-rhythm breathing in a compassionate mind training (CMT) intervention that incorporated principles of CFT. The found that HRV improved for various clinically at risk participants, in fact, some showed clinically significant improvement when they practiced this compassion intervention. Interestingly, Allen and Friedman (2012) found that including a positive emotion focus with 6 breath per minute breathing led to more positive results, particularly for participants uncomfortable breathing difficulty breathing at slow rates. This provides additional evidence that adding compassion focused thoughts and imagery with 6 breath per minute breathing can be beneficial.

Rockliff et al. (2008) along with various other studies show that engagement in self-compassion exercises is positively associated with HRV (Kirby et al., 2017). Whether by decreasing it due to higher levels of self-criticism (Rockliff et al., 2008), increasing HRV resilience to stressful events (Kok et al., 2013), or increasing HRV through compassionate self-talk (Petrocchi et al., 2017), self-compassionate exercises are shown to activate the parasympathetic nervous system, which influences HRV (Kirby et al., 2017). For example, self-critical writing and self-compassionate writing exercises, build from the CFT perspective, were shown to significantly impact HRV levels (Steffen et al., 2020).

### Nature Exposure and Stress Reduction

An alternative approach to physiological regulation used in psychotherapy is nature exposure. A few studies have found that exposure to nature is moderately associated with reduction in stress physiology. In a study by Ulrich et al. (1991), viewing a nature clip increased participants’ parasympathetic response, as demonstrated via decreased blood pressure, skin conductance, muscle tension, and decreased HR. Valtchanov et al. (2010) found that after exposure to a stress condition, participants in the virtual reality nature showed increased positive affect and decreased stress as measured by decreased skin conductance, although HR did not differ between groups. Annerstedt et al. (2013) found that nature sounds led to increased recovery in HR HRV after stressful stimuli.

Blum et al. (2019) administered HRV biofeedback to participants in a standard biofeedback condition and in a virtual reality enhanced nature biofeedback condition. Participants practiced slow diaphragmatic breathing guided by an auditory pacer set at 6 breaths per minute. There were no differences found in physiological measures of stress. In a similar study, Rockstroh et al. (2019) used both traditional biofeedback and virtual reality enhanced nature biofeedback in two conditions that proved to be equally effective in increasing HRV.

### The Present Study

No studies to date have empirically compared breathing at 6 breaths per minute with soothing rhythm breathing in their ability to improve HRV. In the present study we compared 6 breaths per minute breathing with soothing rhythm breathing and with a relaxing nature video using a randomized controlled experimental design. After participating in the experimental condition, we had participants engage in self-critical and then self-compassionate writing exercises followed by a recovery period to investigate stress response following the interventions. We examined four hypotheses. First, we hypothesized that each of the three experimental conditions would lead to increased HRV, self-critical writing would lead to lower HRV, and then the self-compassionate writing and recovery period would lead to increased HRV. Second, we hypothesized that the 6 breath per minute breathing and the soothing rhythm breathing conditions would produce higher HRV than the nature video condition, and that the 6 breath per minute breathing would produce higher HRV than the soothing rhythm breathing. Third, we hypothesized that the 6 breath per minute and soothing rhythm breathing conditions would lead to a smaller decline in HRV relative to the nature video condition during the self-critical writing task. Fourth, we hypothesized that clinically at-risk participants, determined by scores on the Depression, Anxiety, and Stress Scale (DASS) questionnaire, would show overall greater changes in HRV when compared with non-clinically at-risk participants.

## Methods

### Participants

A total of 96 participants were recruited for participation in this study through undergraduate psychology courses at the university. Participants first completed an online survey measuring stress and wellbeing and then participated in the experimental portion of the study. Exclusion criteria consisted of current engagement in mental health treatment, biofeedback, or use of medications that impact the cardiovascular system. All participation was voluntary and was approved by the Institutional Review Board. All participants signed an informed consent before going the study and were informed that they could stop participation at any point during the experiment without penalty. Participants were also given the contact information for the university counseling center and informed to contact them should they feel overly stressed or worried. All participants completed the experiment in its entirety.

### Measures

#### The Depression, Anxiety, and Stress Scale

The DASS is a set of three self-report scales designed to measure the negative emotional states of depression, anxiety, and stress (Sinclair et al., 2012). The DASS is composed of 21 items with seven items for each scale. The DASS was designed as a dimensional measure of general depression, anxiety, and stress. Reliability is very good with Cronbach’s alphas of 0.91 for the depression scale, 0.84 for the anxiety scale, and 0.80 for the stress scale (Sinclair et al., 2012). The depression and anxiety subscales were used in this study. The depression scale assesses dysphoria, hopelessness, devaluation of life, self-deprecation, lack of interest/involvement, anhedonia, and inertia. The anxiety scale assesses general symptoms of anxiety including autonomic arousal, situational anxiety, and subjective experience of anxious affect. Subjects are asked to respond on a four-point severity/frequency scale to rate the extent to which they have experienced each state over the past week. Scores for depression and anxiety are calculated by summing the scores for the relevant items. To identify clinically at-risk participants versus not clinically at-risk participants, established norms were used to divide the sample between low to mild symptoms versus moderate to severe symptoms (Sinclair et al., 2012).

#### The Survey of Positive and Negative Experience

The Survey of Positive and Negative Experience (SPANE) is a 12-item questionnaire that assesses positive (six items) and negative (six items) mood and was administered before the study began (Diener et al., 2009). Participants are asked to rate their experience during the past 4 weeks in terms of feeling positive, negative, good, bad, etc. Each question is rated on a five-point scale, with one being “Very Rarely or Never” and five being “Very Often or Always.” The positive and negative mood items have shown good reliability with Cronbach’s alpha 0.87 for positive mood and Cronbach’s alpha of 0.81 for negative mood. The SPANE was used in this study as a manipulation check to evaluate whether the experimental conditions elicited the desired effects and was given at the end of each experimental phase (baseline, breathing, self-critical writing, self-compassionate writing, and recovery phase).

#### Physiological Assessment

Heart rate variability was assessed using a Nexus-4 biofeedback system (MindMedia, Herten, Netherlands). After having the purpose of the study explained and signing consent, participants were instrumented with a respiration belt and a three lead electrocardiogram using Ag/AgCl electrodes placed on their collar bones and on the bottom of the left rib cage. Interbeat interval data were extracted in 5-min segments and cleaned for artifact using the Kubios program and measures of SDNN, HF HRV, LF HRV, and LF/HF ratio were calculated (Tarvainen et al., 2014). Because HF HRV and LF HRV were not normally distributed, the natural logs of the ms^2^ were used. Blood pressure and HR data was collected at the middle and end of each phase of the study using a GE ProCare monitor.

### Procedures

Participants were randomly assigned to either a HRV biofeedback training group (*n* = 33), a soothing rhythm breathing group (*n* = 32), or a nature video control group (*n* = 31). After sitting quietly for 10 min, participants were instructed to either breathe following a breathing pacer set at 6 breaths per minute for 10 min, listen to and following a recording on soothing rhythm breathing done by Paul Gilbert for 10 min, or watch a high-definition coral reef video with relaxing music for 10 min. This was followed by a 5-min period where participants were instructed to write about a time that they felt very self-critical. After the self-critical writing, the participants wrote self-compassionate statements for 5 min, focusing on having a “caring compassionate self.” The experiment ended with a 10-min recovery period where the participants sat quietly. Therefore, each participant was sitting for a total of 40 min as they experienced the different phases of the study.

### Data Analysis

Data was analyzed SPSS (IBM SPSS Statistics, Version 26). Baseline characteristics were analyzed using descriptive statistics and univariate general linear model analyses. The hypotheses were tested using repeated measures analysis of variance. The three experimental conditions of 6 breath per minute breathing, soothing rhythm breathing, and nature video were examined during breathing practice, self-critical writing, self-compassionate writing, and recovery period controlling for baseline levels. For the HRV variables, the last 5 min of each phase was used in the analyses.

## Results

### Baseline Characteristics by Condition

The baseline characteristics of the participants by condition is presented in Table 1. The participants did not significantly differ by age, gender or ethnic composition, or mood levels at baseline. Measures of HRV (SDNN, HF HRV, and LF HRV) and systolic blood pressure (SBP) were roughly equivalent at baseline. Diastolic blood pressure (DBP) and HR at baseline, however, were highest in the Nature Video condition and lowest in the Soothing Rhythm condition. Depressive symptoms were highest in the 6 breaths per minute breathing condition. We also examined correlations among the HRV and breathing rate measures and measures of mood and depression, anxiety, and stress. None of these relationships were significant.

**TABLE 1:** Baseline characteristics by condition.

	Condition			
	6 breaths/min mean (SD) or % (*n* = 31)	Soothing rhythm mean (SD) or % (*n* = 32)	Nature video mean (SD) or % (*n* = 33)	*p*
Age	21.3 (4.1)	20.5 (2.3)	21.03 (4.2)	0.71
Gender (% female)	73%	67%	69%	0.87
Ethnicity (% white)	93%	87%	88%	0.88
Positive mood	24.6 (5.4)	27.0 (6.1)	26.4 (5.6)	0.20
Negative mood	14.6 (3.2)	14.6 (4.4)	13.8 (4.3)	0.62
Depressive Symptoms	5.8 (4.2)	5.7 (4.9)	3.7 (2.9)	0.07
Anxiety symptoms	6.0 (4.9)	4.9 (3.7)	3.8 (3.2)	0.08
Stress symptoms	8.3 (4.6)	5.8 (3.5)	5.9 (3.7)	0.02
Systolic BP	112 (11)	109 (9)	113 (7)	0.15
Diastolic BP	65 (8)	63 (6)	68 (7)	0.01
Heart rate	74 (13)	68 (9)	74 (10)	0.07
SDNN HRV	53.58 (22.96)	57.58 (24.38)	48.25 (21.35)	0.28
HF HRV	6.05 (1.03)	6.87 (1.08)	6.48 (1.03)	0.27
LF HRV	6.88 (0.88)	7.17 (0.76)	6.97 (0.83)	0.25
LF/HF ratio	1.01 (0.11)	1.06 (0.13)	1.07 (0.15)	0.19
Breathing rate	17.8 (3.1)	18.9 (3.2)	18.3 (3.1)	0.49

### Manipulation Check

The Survey of Positive and Negative Experience was administered as a manipulation check to examine whether breathing practice led to improved mood and self-critical writing led to decreased mood. There was a significant overall effect of the experiment on positive (*F* = 99.65, *p* < 0.001) and negative mood (*F* = 86.95, *p* < 0.001). Negative mood significantly decreased during the breathing practice and nature video conditions, increased during self-critical writing, and then ended significantly lower than baseline during self-compassionate writing and recovery. Positive mood significantly decreased during the self-critical writing and then returned to baseline levels during self-compassionate writing. Positive mood did not significantly increase during the breathing practice and nature video conditions, and positive mood during recovery was not significantly different from baseline levels.

There was a significant within-subjects time x condition effect for positive mood (*F* = 2.371, *p* = 0.02) indicating differential positive mood responses. Planned contrasts using baseline as comparison indicated a non-significant trend for the soothing rhythm condition to have a larger increase in positive mood while practicing soothing rhythm breathing (*F* = 2.45, *p* = 0.09). Additionally, both the soothing rhythm and 6 breath conditions showed a larger increase in positive mood moving from self-critical to self-compassionate writing relative to the nature video condition (*F* = 3.32, *p* = 0.04). The within-subjects time x condition analysis for negative mood was not significant (*F* = 1.63, *p* = 0.11) indicating that change in negative mood over time did not significantly differ by experimental group.

### Hypothesis 1: Impact of the Overall Experiment on HRV, Breathing Rate, and BP

The first hypothesis stated there would be an overall experimental effect, with HRV (SDNN, HF HRV, LF HRV, LF/HF ratio) increasing during practice, decreasing during self-critical writing, and then increasing again during self-compassionate writing and the recovery period. Similarly, SBP, DBP, and HR were hypothesized to decrease during practice, increase during self-critical writing, and then decrease again during self-compassionate writing and recovery. Respiration rate was hypothesized to decrease during practice, increase during self-critical writing, and then decrease during self-compassionate writing and the recovery period. Means and standard deviations for these variables are presented in Table 2.

**TABLE 2:** Mean (SD) values for HRV and breathing rate values across the experiment.

	Baseline	Breathing	Self-critical	Self-compassion	Recovery
**SDNN**					
Total	53.1 (22.8)	71.6 (29.8)	49.1 (22.8)	48.8 (23.3)	59.8 (24.5)
6 breath	53.6 (23.0)	84.9 (26.0)	52.4 (26.3)	51.9 (24.9)	61.8 (23.7)
Soothing	57.9 (23.9)	80.2 (26.3)	50.7 (21.3)	49.9 (26.4)	64.5 (25.0)
Nature video	48.3 (21.3)	50.9 (25.3)	44.5 (22.7)	48.8 (23.3)	53.7 (24.4)
**HF HRV**					
Total	6.7 (1.1)	6.8 (1.0)	6.5 (1.2)	6.5 (1.1)	6.9 (1.0)
6 breath	6.9 (1.0)	6.7 (1.0)	6.6 (1.2)	6.6 (1.1)	7.1 (1.0)
Soothing	6.9 (1.1)	7.0 (0.9)	6.5 (1.2)	6.5 (1.3)	7.0 (1.1)
Nature video	6.5 (1.0)	6.6 (1.0)	6.3 (1.3)	6.3 (1.0)	6.7 (0.8)
**LF HRV**					
Total	7.0 (0.8)	7.8 (1.2)	6.9 (0.8)	6.9 (1.1)	7.3 (0.9)
6 breath	6.9 (0.9)	8.4 (0.8)	7.0 (0.9)	6.9 (0.8)	7.3 (0.9)
Soothing	7.2 (0.7)	8.2 (0.8)	7.0 (0.8)	6.8 (1.5)	7.5 (0.09)
Nature video	6.9 (0.8)	6.8 (1.1)	6.8 (0.8)	6.7 (0.8)	7.1 (1.0)
**LF/HF ratio**					
Total	1.1 (0.1)	1.2 (0.2)	1.1 (0.2)	1.1 (0.1)	1.1 (0.1)
6 breath	1.0 (0.1)	1.3 (0.2)	1.1 (0.1)	1.1 (0.1)	1.1 (0.1)
Soothing	1.1 (0.1)	1.2 (0.2)	1.1 (0.2)	1.1 (0.2)	1.1 (0.1)
Nature video	1.1 (0.2)	1.0 (0.1)	1.1 (0.2)	1.1 (0.1)	1.1 (0.1)
**Breathing rate**					
Total	18.3 (3.1)	14.3 (4.4)	19.1 (3.1)	19.0 (3.2)	15.9 (3.6)
6 breath	17.8 (3.2)	12.2 (4.3)	19.3 (3.4)	19.4 (3.3)	14.8 (3.7)
Soothing	18.7 (3.2)	13.1 (4.2)	19.1 (3.1)	18.7 (3.5)	16.3 (3.8)
Nature video	18.3 (3.1)	16.9 (3.5)	19.0 (3.0)	19.0 (3.2)	16.4 (3.3)

SDNN, LF HRV, and the LF/HF ratio significantly increased during practice, decreased during self-critical writing, and then increased again during the self-compassionate writing and recovery periods (*F* = 55.40, *p* < 0.001; *F* = 38.59, *p* < 0.001; *F* = 19.43, *p* < 0.001). HF HRV, however, did not significantly change during practice, but significantly decreased during self-critical writing and stayed low during self-compassionate writing, and then significantly increased during the recovery period (*F* = 14.47, *p* < 0.001).

Breathing rate significantly decreased during breathing practice, returned to baseline levels during self-critical and self-compassionate writing, and ended at lower than baseline levels during recovery (*F* = 95.65, *p* < 0.001). Participants were novices not used to breathing at 6 breaths per minute. During breathing practice, the 6 breath per minute condition decreased from 17.8 at baseline to 12.2 during practice on average, the soothing rhythm condition decreased from 18.7 at baseline to 13.1 during practice on average, and the nature video control condition decreased from 18.3 to 16.9 during the video on average.

SBP and DBP significantly decreased during practice, returned to baseline levels during self-critical writing, and then decreased to lower than baseline during recovery (*F* = 5.27, *p* < 0.001; *F* = 6.13, *p* < 0.00). HR significantly increased during practice, stayed high during critical and self-compassionate writing, and then significantly decreased to below baseline levels during recovery (*F* = 19.55, *p* < 0.001).

### Hypothesis 2: Impact of Condition on HRV, Breathing Rate, and BP

**FIGURE 1:**
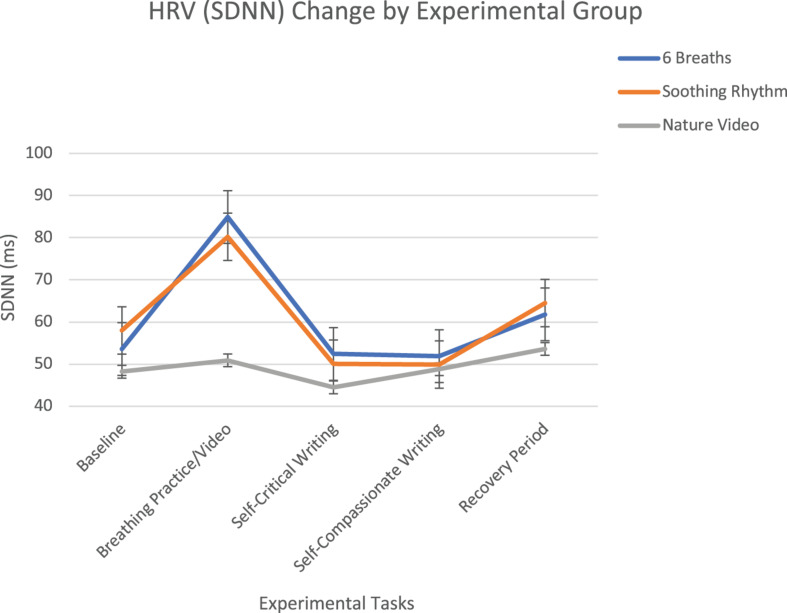
SDNN change over experimental tasks by experimental group with standard error bars. The 6 breath per minute and soothing rhythm breathing conditions both increased significantly during the breathing practice, whereas the nature video did not change.

The second hypothesis stated the 6 breath per minute condition would exert a stronger influence on HRV and BP than the soothing rhythm and nature video conditions, and that the soothing rhythm condition would exert a stronger influence on HRV and BP than the nature video condition. There was a significant within-subjects time *x* condition effect for SDNN (*F* = 8.64, *p* < 0.001). Planned contrasts using baseline as comparison found that SDNN increased significantly during practice for the 6-breath and soothing rhythm conditions, but did not change in the nature video condition (*F* = 19.61, *p* < 0.001) (Figure 1). The conditions were not significantly different in response to writing or during recovery. There was no within-subjects time x condition effect of HF HRV (*F* = 0.96, *p* = 0.47), with none of the conditions showing an HF HRV response during practice. HF HRV did decrease significantly during the self-critical writing and then returned to baseline levels during the recovery phase.

**FIGURE 2:**
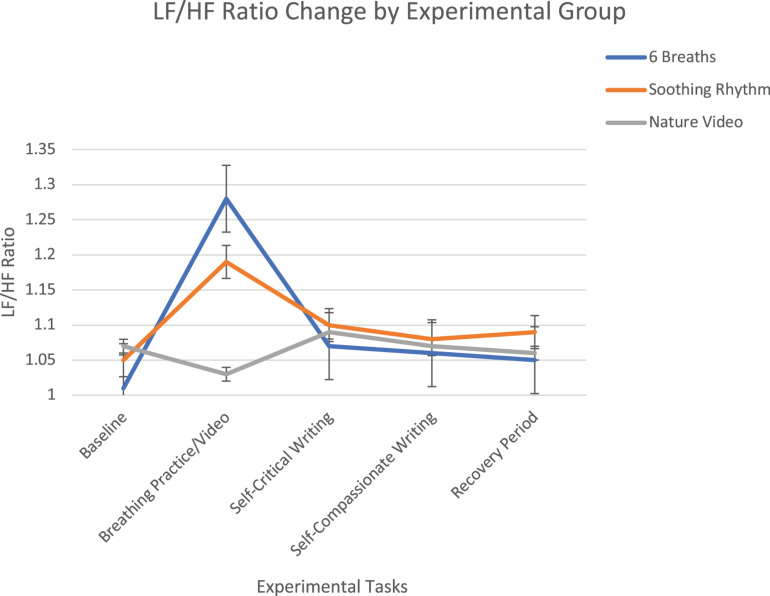
LF/HF ratio change over experimental tasks by experimental group with standard error bars. The 6 breath per minute and soothing rhythm breathing conditions both increased significantly during the breathing practice, whereas the nature video did not change, and the 6 breath per minute group increased significantly more than the soothing rhythm breathing.

There was a significant within-subjects time *x* condition effect for LF HRV (*F* = 10.03, *p* < 0.001). Planned contrasts using baseline as comparison found that LF increased significantly during practice for both the 6-breath and soothing rhythm breathing conditions, with LF increasing more for the 6-breath condition then the soothing rhythm condition, with LF not changing during the nature video condition (*F* = 35.50, *p* < 0.001). The conditions were not significantly different in response to writing or during recovery. There was a significant time x condition effect for the LF/HF ratio (*F* = 12.41, *p* < 0.001). Planned contrasts using baseline as comparison found that both 6-breath and soothing rhythm breathing conditions significantly increased from baseline more than the nature video condition, and the 6 breath per minute condition increased significantly more than the soothing rhythm breathing condition (*F* = 24.68, *p* < 0.001; see Figure 2). The conditions were not significantly different in response to writing or during recovery. Overall, 6 breath per minute breathing resulted in significantly higher LF HRV and LF/HF ratio levels than the soothing rhythm breathing, and the nature video conditions, and soothing rhythm breathing resulted in significantly higher LF HRV and LF/HF ratio levels than the nature video condition.

Breathing rate significantly decreased for the 6 breaths per minute condition and the soothing rhythm condition during breathing practice but did not change for the nature video (*F* = 9.04, *p* < 0.001) and the three conditions were not significantly different during writing or during recovery. There were no time *x* condition effects for SBP (*F* = 1.08, *p* = 0.38), DBP (*F* = 1.13, *p* = 0.35) or HR (*F* = 0.63, *p* = 0.76).

### Hypotheses 3 and 4: Recovery and the Impact of Depressive and Anxious Depressive Symptoms

The third hypothesis stated that the 6-breath per minute and soothing rhythm breathing conditions would show a smaller decline in HRV relative to the nature video control condition during self-critical writing. Planned contrasts using baseline as comparison found no significant differences, however, among conditions for SDNN (*F* = 1.58, *p* = 0.21), LF HRV (*F* = 1.38, *p* = 0.26), or the LF/HF ratio (*F* = 1.36, *p* = 0.34). The experimental conditions did not differ in their HRV response to the writing or recovery conditions. The fourth hypothesis stated that clinically at-risk participants who scored in the moderate to severe ranges on depressive or anxious symptoms would be more responsive to the breathing training and to the writing conditions. There were no significant differences, however, for lower versus higher symptoms for depression or anxiety (SDNN: *F* = 1.15, *p* = 0.33; HF HRV: *F* = 0.01, *p* = 0.99; LF HRV: *F* = 1.12, *p* = 0.29). Participants responded similarly to the breathing training and writing conditions regardless of level of symptoms.

## Discussion

Our first hypothesis was that each of the experimental conditions, 6 breaths per minute breathing, soothing rhythm breathing, and watching a nature video, would lead to increased SDNN, HF HRV, LF HRV, and LF/HF ratio relative to baseline. For SDNN, LF HRV, and the LF/HF ratio, only the 6 breath per minute and soothing rhythm breathing conditions showed a significant increase from baseline. None of the conditions had a significant impact on HF HRV. It is important to note that the participants 6 breath per minute pacer condition were unable to consistently breathe at that rate, instead achieving an average pace of 12 breaths per minute. The self-critical writing had a potent effect, significantly decreasing SDNN, HF HRV, LF HRV, and the LF/HF ratio, and these parameters returned to baseline levels by the recovery period. Our second hypothesis was that the 6 breath per minute condition would lead to increased HRV relative to the other conditions. The 6 breath per minute and soothing rhythm conditions did not differ in SDNN response, although the 6 breath per minute group did have a significantly larger LF HRV and LF/HF ratio response. The soothing rhythm breathing condition was significantly better than the nature video on improving LF HRV and the LF/HF ratio.

The third hypothesis stated that the 6 breath per minute and soothing rhythm conditions would show increased HRV recovery during the writing tasks and recovery. There were not differences found, however, among the conditions. The fourth hypothesis was that clinically at-risk participants, as measured by moderate to severe symptoms on the DASS, would show a smaller HRV responses during the self-critical writing condition. However, both the clinically at-risk and the non-clinically at-risk participants showed roughly equal responses.

Our lack of findings for HF HRV is in line with the Lin et al. (2019) biofeedback plus CBT study that found increases in SDNN, LF HRV, and the LF/HF ratio but no differences in HF HRV. Van Diest et al. (2014) similarly found decreased HF for those breathing at 6 breaths per minute compared to those breathing 12 breaths per minute. In a recent review of HRV for breathing 6 breaths per minute, Schwerdtfeger et al. (2020) concluded that this breathing pattern tends to have little effect on HF HRV. This makes sense for our results with the 6 breath per minute condition, which takes a similar approach to what occurs in the HRV biofeedback that Lin et al. (2019) and Van Diest et al. (2014) used. The lack of findings for HF HRV, however, does not fit with previous studies on soothing rhythm breathing (Chien et al., 2015; Russo et al., 2017; Li et al., 2018). It appears that one session of practice was insufficient to match what was seen in previous studies.

The 6 breath per minute breathing condition led to the largest increases in LF HRV and LF/HF ratio. This is in line with previous studies finding that breathing at resonance frequency (about 6 breaths per minute) leads to a spike in the HRV frequency spectrum at 0.1 Hz, which is in the middle of the LF range (Lehrer and Gevirtz, 2014; Nagarajan, 2014; Shaffer et al., 2014). Bernston et al. (1997) note that “when respiration rate is lower than about 10 breaths/min” the “broader bandwidth allows the 0.1 Hz rhythm to be contaminated by respiratory sinus arrythmia (RSA)” (p. 637), and that “RSA can be substantially greater during slow than during faster breathing” (p. 627), so 6 breath per minute breathing is extending the range at which we see the impact of RSA. This relationship between RSA and breathing rate has been known since as early as the 1960s when Angelone and Coulter (1963) found that breathing at about 6 breaths per minute lead to the highest changes in HRV.

Interestingly, the nature video control condition did not impact any of the HRV variables. As noted previously, exposure to nature and to nature videos has been related to improvements in HRV in some studies but not all (Ulrich et al., 1991; Blum et al., 2019; Rockstroh et al., 2019). A nature video was used as a control because it is simple to sit and watch a video; if it has a positive effect, therapists can simply recommend watching a video to help manage physiological reactivity to mood and stress. While there is some evidence for the benefits of nature videos, in this study the 6 breaths per minute and soothing rhythm breathing conditions had significantly more impact on HRV, indicating that breathing training is worth the effort.

For those interested in addressing physiological regulation in psychotherapy, the main implication of this study is that both 6 breath per minute breathing and soothing rhythm breathing increase HRV and therefore be beneficial to use in psychotherapy. For those interested in developing resonance or coherence, which occurs in the LF range, 6 breath per minute breathing demonstrated larger effects than soothing rhythm breathing. The findings of this study also support using self-critical writing and self-compassionate writing as a method to induce changes in HRV. Self-critical writing in particular resulted in a step decrease in HRV followed by a slow recovery.

### Limitations and Future Directions

There are several limitations to keep in mind in interpreting these findings. First, the experiment consisted of one session of breathing practice. It is not known what effect multiple sessions of practice will have on HRV outcome. For example, it is possible that more practice over time would have a positive impact on HF HRV as seen in some studies. Second, all participants were young healthy college students, so it is not clear if these results would be seen in older participants or those with health conditions. Third, the breathing training was not embedded in a psychotherapy session so it is not clear if the results would be similar in that context. In this study, depressive and anxious symptoms were measured and examined, but symptoms were not related to HRV at baseline or in response to the various conditions. Fourth, no dietary restrictions were given for the study and what participants ate or drank before participating was not assessed so we were not able to control for those variables. Future studies could build on these findings by examining these different breathing techniques over time and in psychotherapy settings, and by examining older participants.

## Conclusion

Two breathing techniques that have been used in psychotherapeutic contexts, 6 breath per minute breathing and soothing rhythm breathing, significantly improved the time domain measure (SDNN) of HRV, whereas the control condition of watching a nature video did not impact HRV. LF HRV and LF/HF ratio were significantly higher in the breathing conditions relative to watching the nature video, and the 6 breath per minute condition resulted in higher LF HRV and LF/HF ratio than the soothing rhythm condition. However, the participants in the 6 breathe per minute pacer condition were not able to consistently breath at 6 breathes per minute, possibly indicating the importance of regular practice to obtain this rate. In this study, HF HRV was not impacted by either breathing condition or by watching a nature video. Overall, evidence supports using breathing techniques to help regulate physiological functioning. For those interested in developing resonance or coherence specifically in breathing, the 6-breath condition appears the best choice. Given the initial positive findings in this study and in previously published studies there appears to be great benefit in integrating empirically tested breathing techniques into therapy.

## Data Availability Statement

The raw data supporting the conclusions of this article will be made available by the authors, without undue reservation.

## Ethics Statement

The studies involving human participants were reviewed and approved by BYU Institutional Review Board. The patients/participants provided their written informed consent to participate in this study.

## Author Contributions

PS conceptualized, designed, and supervised study, analyzed the data, and participated in writing each section. DB and RC helped design and conduct study, and ran participants. KJ, MC, and JB helped conduct study, recruited and ran participants, and participated in writing each section. MP helped design and conduct study, and ran participants. All authors contributed to the article and approved the submitted version.

## Conflict of Interest

The authors declare that the research was conducted in the absence of any commercial or financial relationships that could be construed as a potential conflict of interest.
